# Editorial: Public and community engagement in health science research: Openings and obstacles for listening and responding in the majority world

**DOI:** 10.3389/fpubh.2022.1012678

**Published:** 2022-08-31

**Authors:** Gillian F. Black, Phaik Yeong Cheah, Mary Chambers, Deborah Nyirenda

**Affiliations:** ^1^Sustainable Livelihoods Foundation, Cape Town, South Africa; ^2^Mahidol Oxford Tropical Medicine Research Unit, Faculty of Tropical Medicine, Mahidol University, Bangkok, Thailand; ^3^Nuffield Department of Medicine, Centre for Tropical Medicine and Global Health, University of Oxford, Oxford, United Kingdom; ^4^Oxford University Clinical Research Unit, Hospital for Tropical Diseases, Ho Chi Minh City, Vietnam; ^5^Malawi Liverpool Wellcome Trust Clinical Research Programme, Community Engagement and Bioethics, Blantyre, Malawi

**Keywords:** community engagement, health science research, participation, co-production, resource-limited settings

Community engagement is recognized as a valuable and ethical component of health science research and its inclusion is increasingly becoming a prerequisite for research funding and approvals (1–5). In general terms, community engagement aims to foster the interchange of perspectives, opinions, and ideas and promote the co-production of knowledge between researchers, research participants, and other stakeholders (6). Community engagement initiatives are often designed with the intention of enabling exchanges of this nature.

This Research Topic was designed to explore approaches taken by engagement practitioners, engagement scholars, social scientists, and researchers to promote listening and responding to community voices in research processes. It seeks to understand the challenges that obstruct meaningful integration of community voices in research design and responsiveness to expressions of needs and aspirations for change, in low-and-middle-income countries. The Research Topic draws experience from numerous majority world countries and explores multiple global health challenges and research approaches. The majority world is “where the vast majority of the world's people live yet they have access to a fraction of the world's wealth and power” (7). By discussing projects, programmes or guidelines, each article provides valuable experience and insight into the effectiveness of efforts to promote listening and responsiveness in community engagement initiatives. The Research Topic comprises 10 articles including six original research papers, two community case studies, one methods article, and one perspective piece. Experiences are shared from Southeast Asia, Africa, and South America.

The first six articles discuss approaches and methods suggested or used to engage community members in pressing public health challenges and ethically complex fields of research.

The perspective article by Hickey et al. draws on data collected as part of an evaluation of community and public engagement (CPE) by National Institute of Health Research (NIHR) award holders to provide insights on CPE practice in global health research. The authors build on their analysis of this data and existing guidance to identify key components of “good” CPE.

Quoc et al. describe the methods and results of a situation analysis undertaken as part of community-based participatory research (CBPR) to engage southern Vietnamese communities in discussions about access to care for hepatitis C virus (HCV). The authors aimed to identify key groups and institutions working with underserved populations that are at high risk of HCV infection including people who inject drugs and those with limited resources (often migrant workers). The article emphasizes the value of using stakeholder information to build relationships, foster ownership, and ensure context specificity in CBPR.

In northern Vietnam, Cai et al. developed a participatory learning and action intervention that used community-led photography to address the problem of antimicrobial resistance (AMR) among both humans and animals. The intervention was implemented in preparation for a large-scale One Health trial. Through the thematic analysis of implementation documentation, the article shares important lessons learned in relation to optimizing participatory AMR engagement strategies that can add value to the conceptualization and design of community engagement activities.

Another participatory visual methods (PVM) approach to engagement in Southeast Asia is discussed by Delmas et al. The authors describe the development of a script for a film that was designed to engage thousands of community members living along the Thai-Myanmar border on the highly prevalent health challenge of tuberculosis. Their research shows that locally made films, which include patients and community members in script development and as leading actors, can have a significant impact on various aspects of disease awareness and knowledge.

Moving to an African context, Davies et al. also discuss the use of visual methods, in their case for the combined purpose of engagement and evaluation. This article focuses on the application of participatory video (PV) to explore the influence of a School Engagement Programme on the views and understandings of science and research among Kenyan state secondary school students. The authors draw on insights gained through facilitating the PV process to make recommendations for school engagement practice.

The case study published by Mumba et al. was also undertaken in Kenya. The authors discuss their experience of community and stakeholder engagement in human infection studies (HIS). They explain that HIS are complex because they involve infecting healthy individuals with disease-causing pathogens which can raise community concerns and jeopardize trust. The article describes how engagement activities were facilitated throughout a controlled human malaria infection study, highlighting the need for guidelines addressing specific considerations of HIS engagement.

The Research Topic also applies a critical lens to engagement frameworks and outcomes by discussing constraints in researcher, community, and government responsiveness.

Polidano et al. discuss their model of decolonial community engagement in a global health research program, focusing on cutaneous leishmaniasis. Their methodology implied that models for community engagement would be different in the culturally diverse contexts of Brazil, Ethiopia, and Sri Lanka. The authors evaluate their critical anthropological approach to engagement and in doing so reveal a gap between the exemplary community engagement frameworks available in the literature and the everyday reality of working in low-resourced communities.

Similar conclusions are drawn by Black and Sykes as they share insights from South Africa. The authors describe a case study of community engagement in water microbiology undertaken during a water crisis in Cape Town and the encroaching threat of a “Day Zero” when piped water supplies would be shut off (see Figure 1). They introduce the concept of engagement integrity to depict the gap between recommended standards of engagement formulated by global health organizations and what is achievable in marginalized contexts characterized by structural deficits and political exclusion.

**Figure 1:**
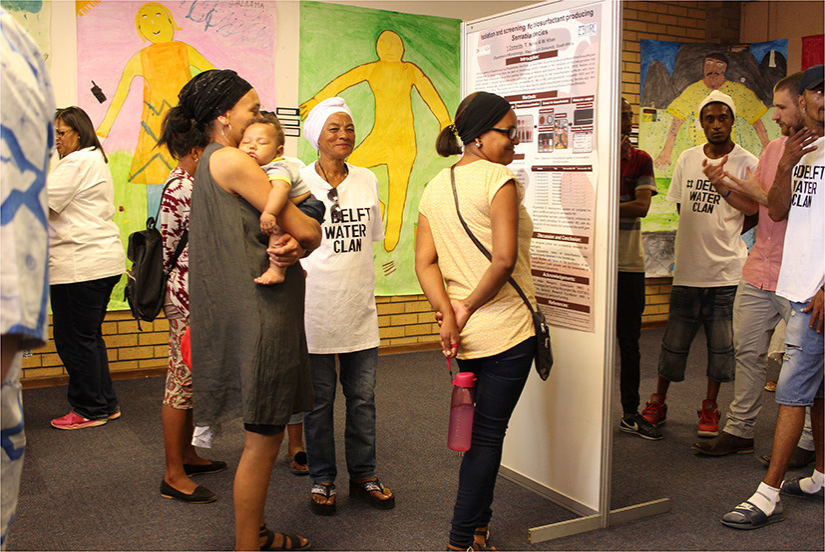
A public exhibition held as part of an engagement project in water microbiology undertaken during a water crisis in Cape Town described by Black and Sykes.

The article by Nyirenda et al. raises questions about the opportunity for participatory community engagement to foster social justice in settings with pronounced social and structural inequalities. The authors report that digital story telling was an effective method for engaging community members in self-identified priority health challenges related to water, sanitation, and hygiene in urban Malawi. They go on to discuss how a lack of resources and power imbalances prevented participants from escalating their dissatisfaction through community activism.

Nouvet et al. examine responses to the question “*Is There Anything Else You Would Like to Add?*” in the context of a study that explored perceptions of Ebola research among West Africans. The authors raise important questions about what can and should be done when concerns and hopes expressed by research participants exceed the intended scope of a research project and ask what is at stake ethically in how researchers respond to such entreaties.

Collectively, the articles in this Research Topic share significant obstacles encountered, and valuable lessons learned through the design, implementation, and assessment of community engagement initiatives. By drawing on their learning the authors raise important questions and offer recommendations with the intention of strengthening and grounding community engagement practice in global health research in resource-limited contexts.

## Author contributions

The authors of this article were co-guest editors of the Research Topic. GB wrote the first draft of the editorial. PYC, DN, and MC reviewed and provided feedback on the manuscript. All authors contributed to the article and approved the submitted version.

## Conflict of interest

Author GB was employed by Sustainable Livelihoods Foundation. The remaining authors declare that the research was conducted in the absence of any commercial or financial relationships that could be construed as a potential conflict of interest.

## Publisher's note

All claims expressed in this article are solely those of the authors and do not necessarily represent those of their affiliated organizations, or those of the publisher, the editors and the reviewers. Any product that may be evaluated in this article, or claim that may be made by its manufacturer, is not guaranteed or endorsed by the publisher.
